# Prediction and bioactivity of small-molecule antimicrobial peptides from *Protaetia brevitarsis* Lewis larvae

**DOI:** 10.3389/fmicb.2023.1124672

**Published:** 2023-03-16

**Authors:** Qian Fu, Dengtian Cao, Jing Sun, Xinbo Liu, Haitao Li, Changlong Shu, Rongmei Liu

**Affiliations:** ^1^College of Life Sciences, Northeast Agricultural University, Harbin, China; ^2^State Key Laboratory for Biology of Plant Diseases and Insect Pests, Institute of Plant Protection, Chinese Academy of Agricultural Sciences, Beijing, China

**Keywords:** *Protaetia brevitarsis* Lewis larvae, AMPs, small molecule design, antimicrobial activity, APD3, truncated sequence

## Abstract

Antimicrobial peptides (AMPs) are widely recognized as promising natural antimicrobial agents. Insects, as the group of animals with the largest population, have great potential as a source of AMPs. Thus, it is worthwhile to investigate potential novel AMPs from *Protaetia brevitarsis* Lewis larvae, which is a saprophagous pest prevalent in China. In this study, comparing the whole-genome sequence of *Protaetia brevitarsis* Lewis larvae with the Antimicrobial Peptide Database (APD3) led to the identification of nine peptide templates that were potentially AMPs. Next, based on the peptide templates, 16 truncated sequences were predicted to the AMPs by bioinformatics software and then underwent structural and physicochemical property analysis. Thereafter, candidate small-molecule AMPs were artificially synthesized and their minimal inhibitory concentration (MIC) values were assessed. A candidate peptide, designated FD10, exhibited strong antimicrobial activity against both bacteria and fungi comprising *Escherichia coli* (MIC: 8 μg/mL), *Pseudomonas aeruginosa* (MIC: 8 μg/mL), *Bacillus thuringiensis* (MIC: 8 μg/mL), *Staphylococcus aureus* (MIC: 16 μg/mL), and *Candida albicans* (MIC: 16 μg/mL). Additionally, two other candidate peptides, designated FD12 and FD15, exhibited antimicrobial activity against both *E. coli* (MIC: both 32 μg/mL) and *S. aureus* (MIC: both 16 μg/mL). Moreover, FD10, FD12, and FD15 killed almost all *E. coli* and *S. aureus* cells within 1 h, and the hemolytic effect of FD10 (0.31%) and FD12 (0.40%) was lower than that of ampicillin (0.52%). These findings indicate that FD12, FD15, and especially FD10 are promising AMPs for therapeutic application. This study promoted the development of antibacterial drugs and provided a theoretical basis for promoting the practical application of antimicrobial peptides in the *Protaetia brevitarsis* Lewis larvae.

## 1. Introduction

As one of the greatest inventions of the last century, antibiotics are widely used in the treatment of infectious diseases (Durand et al., 2019). However, antibiotic resistance has become a key concern, largely due to the natural selection of resistant bacteria in response to irrational use of antibiotics (Frieri et al., 2017). The World Health Organization has been concerned about the problems of antibiotic resistance. These issues have been identified as highly serious challenges to public health and sustainable health care (Manniello et al., 2021). Therefore, there is an urgent need to find ways to tackle antibiotic resistance.

Antimicrobial peptides (AMPs) are a potential alternative to antibiotics due to their broad-spectrum antimicrobial activities and low drug resistance (Zhang et al., 2019), and they exhibit broad biomedical application prospects on account of their anticancer, antiviral, antiparasitic, and immunomodulatory effects (Shi et al., 2021). According to the site of action, AMPs are divided into two categories: membrane and non-membrane disruption AMPs. The former increases the permeability of phospholipid bilayers and leads to cell death. The latter may interact with intracellular macromolecules and eventually kill cells (Thakur et al., 2022). Regardless of the mechanism of action, AMPs must proceed through four steps: (1) attract, i.e., AMPs are attracted to the surface of microbes by electrostatic interaction; (2) enrichment, i.e., AMPs replace cations on the cell membrane and cause outer membrane lysis; (3) combination, i.e., AMPs interact with the lipid membrane; and (4) AMP insertion and membrane permeation followed by the AMPs killing cells in a variety of ways (Yeasmin et al., 2021). At present, five models explaining membrane permeabilization are widely accepted: carpet model (Dean et al., 2010), barrel stave model (Ferreira Cespedes et al., 2012), aggregate channel model (Wu et al., 1999), toroidal model (Bozelli et al., 2012), and detergent-like model (Bechinger and Lohner, 2006). To date, the sources of AMPs are extremely broad (Di Somma et al., 2020). The Antimicrobial Peptide Database (APD) shows that most AMPs are derived from animals, while only a few are from plants and bacteria. Therefore, insects, as the group of animals with the largest population, have great potential as a source of AMPs (Sahoo et al., 2021).

Insect AMPs are small cationic molecules with various biological activities, and they are an essential component of the insect innate immune system (Wu et al., 2018). According to their structure and function, insect AMPs can be classified into four categories: comprising α-helical (cecropin and moricin), cysteine-rich (defensin and drosomycin), proline-rich (apidaecin and lebocin), and glycine-rich (attacin and glover) peptides (Yi et al., 2014). Cecropins are made up of two α-helical structures, and they exhibit antimicrobial activity against both Gram-positive and Gram-negative bacteria (Peng et al., 2019). Insect defensins are composed of α-helix and β-pleated structures, and they mainly exhibit antimicrobial activity against Gram-positive bacteria (Koehbach, 2017). Proline-rich peptides consist of 15–34 amino acids, and they exhibit antimicrobial activity against both Gram-positive and Gram-negative bacteria (Rayaprolu et al., 2010; Rao et al., 2012; Armas et al., 2021). Glycine-rich peptides mainly exhibit antimicrobial activity against Gram-negative bacteria (Mrinal and Nagaraju, 2008; Moreno-Habel et al., 2012). Currently, there are more than 300 insect AMPs listed in the APD, including from Coleoptera (Manniello et al., 2021; Xia et al., 2021), Diptera (Aittomaki et al., 2017; Gong et al., 2022), Hemiptera (Nishide et al., 2022), and Lepidoptera (Chowdhury et al., 2020).

*Protaetia brevitarsis* Lewis (Coleoptera: Scarabaeidae) is an important agricultural pest distributed in most areas of China. Its larvae are saprophytic, which means that they are exposed to many different pathogenic microorganisms in the living environment (Wang et al., 2019), so their immune system secretes AMPs. In 2003, three AMPs from *P. brevitarsis* Lewis, designated protaetins 1, 2, and 3, were purified and characterized; protaetin 2 was shown to have activity against Gram-positive and Gram-negative bacteria (Yoon et al., 2003). In 2007, Yoo et al. (2007) extracted fatty acids with anticancer activity from *P. brevitarsis* Lewis. In addition, Kang et al. (2012) demonstrated that a *P. brevitarsis* Lewis larval extract exhibits potent hepatoprotective effects. Later, Lee et al. (2014) found that *P. brevitarsis* Lewis larval extracts exhibit hepatoprotective and antineoplastic properties. However, despite the many studies on the control of *P. brevitarsis* Lewis, there are few studies on its medicinal value, though it has important research value as a kind of medicinal insect.

**Figure 1:**
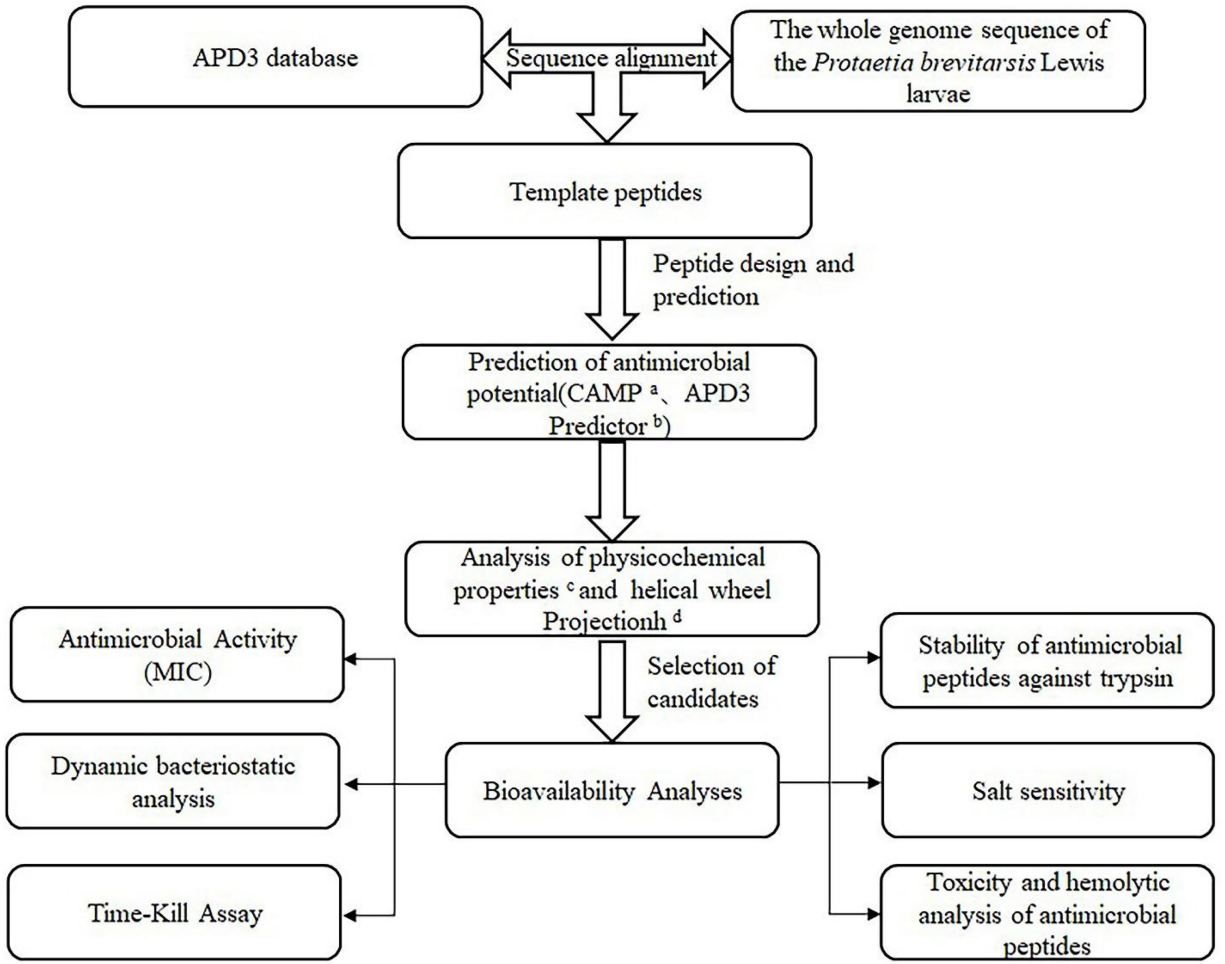
Strategistic diagram of the methodology used to select potential antibacterial peptides in the *P. brevitarsis* Lewis. ^a^CAMP (http://www.camp.bicnirrh.res.in/). ^b^APD3 Predictor (http://aps.unmc.edu/AP/prediction/prediction_main.php). ^c^ExPASy–ProtParam (http://web.expasy.org/protparam/). ^d^Heliquest (https://heliquest.ipmc.cnrs.fr/).

In our laboratory, *P. brevitarsis* Lewis was subjected to whole-genome sequencing (unpublished data). By comparing the whole-genome sequence with the APD, it was found that some *P. brevitarsis* Lewis gene sequences corresponded to AMP sequences. To obtain highly active small-molecular-weight AMPs, truncation, amino acid substitutions, and N-terminal acetylation were used to modify the AMP sequences. Thereafter, their antimicrobial activity, hemolytic effects, cytotoxicity, and stability were studied *in vitro*. This study could provide ideas for the design of small-molecule AMPs and contribute to solutions for the issue of antibiotic resistance. To increased awareness of this study, it was illustrated with the strategistic diagram (Figure 1).

## 2. Materials and methods

### 2.1. Microbial strains and cell lines

The bacterial strains *Staphylococcus aureus* (*S. aureus*) CMCC(B) 26003 and *Pseudomonas aeruginosa* (*P. aeruginosa*) CMCC(B)10104, and the fungal strain *Candida albicans* (*C. albicans*) CMCC(F) 98001, were purchased from Beijing Kezhan Biotechnology Co., Ltd. 2019, China. The bacterial strains *Bacillus thuringiensis* and *Escherichia coli* were obtained from our laboratory. Mouse RAW 264.7 macrophages were purchased from Shanghai Yaji Biotechnology Co., Ltd. 2022, China.

### 2.2. Reagents

Mueller–Hinton broth (MHB), Mueller–Hinton agar (MHA), nutrient agar (NA), and Sabouraud dextrose agar (SDA) were obtained from BEIJING AOBOXING BIO-TECH Co., Ltd. 2019, China. Trypsin was obtained from Nanjing Beyotime Biotechnology Co., Ltd., 2021. 3-(4,5-dimethylthiazol-2-yl)-2,5-diphenyltetrazolium bromide (MTT) was purchased from Sigma-Aldrich, 2021 (China).

### 2.3. Preparation of microbial suspensions

*Staphylococcus aureus* CMCC(B) 26003, *B. thuringiensis*, *E. coli*, and *P. aeruginosa* CMCC(B)10104 were cultured in NA medium and *C. albicans* CMCC(F) 98001 was cultured in SDA medium. They were incubated in a constant-temperature (bacteria: 37°C; fungi: 28°C) incubator for 16–20 h. Thereafter, single colonies were picked and incubated in 50 mL MHB at 160 rpm/min at the optimal growth temperature (bacteria: 37°C; fungi: 28°C). Microbial suspensions of 1 × 10^6^ CFU/mL were then prepared (Chen et al., 2016).

### 2.4. Screening for template peptides

The whole-genome sequence of *Protaetia brevitarsis* Lewis larvae (GenBank accession number: CM014285.1) was compared to the AMP sequences in APD3.^1^ Aligned sequences were used as template peptide sequences (Houyvet et al., 2018).

### 2.5. Predicted AMPs

The template peptide was used to screen out the truncated sequences, and then the antimicrobial region within the predicted peptide was screened using the random forest classifier calculation method in CAMP^2^ to predict the possibility of becoming AMPs. Thereafter, the obtained truncated candidate peptides were inputted into Antimicrobial Peptide Predictor^3^ in APD3 for prediction of the probability of being the truncated sequence of an AMP. The physicochemical properties of the peptides were analyzed by using the ExPASy website software ProtParam^4^ in order to select peptides with a high electrostatic charge, high hydrophobic amino acid ratio, high stability, CAMP prediction of high probability of being an AMP, and APD3 prediction of high probability of being the truncated sequence of an AMP (Yang et al., 2018).

In addition, depending on the structure–function relationships relevant to each AMP, the peptide was selectively modified by amino acid substitution or N-terminal acetylation. For example, negatively charged amino acid residues could be replaced with positively charged amino acid residues (ANWDKVIR changed to ANWKKVIR) and -NH_2_ could be added to the end of the peptide (STLHLVLRLR-NH_2_; STLHLVLRLR) to preserve its functional activity (Chen et al., 2016; Yang et al., 2018). The 16 designed peptides were sent to Beijing Coolaber Technology Ltd. (2020). for chemical synthesis.

### 2.6. Preliminary screening of 16 candidate peptides for antimicrobial activity by agar punch method

100 μL of 1 × 10^6^ CFU/mL microbial suspension (*E. coli*, *P. aeruginosa* CMCC(B)10104, *B. thuringiensis*, *S. aureus* CMCC(B) 26003, or *C. albicans* CMCC(F) 98001) was evenly applied to dishes containing 20 mL NA (bacteria) or SDA (fungi). After the microbial solution solidified, six holes per dish were punched with a sterile pipette tip of 6 mm in diameter, with each hole spaced >20 mm apart and > 10 mm apart from the edge of the dish. The holes were marked with (+), (−), and (1–16), representing the positive control (ampicillin), negative control (deionized water), and treatment groups (16 candidate peptides dissolved in sterile water), respectively. Next, 55 μL of 1 mg/mL control/treatment was added to each hole and incubated in a constant-temperature (bacteria: 37°C; fungi: 28°C) incubator for 16–18 h (Chen et al., 2016; Gong et al., 2022), with three replicates for each treatment group. The diameter of the inhibition circle was measured using ImageJ software (National Institutes of Health, United States) in order to preliminarily determine whether the candidate peptides had inhibitory activity.

### 2.7. Minimal inhibitory concentration (MIC) of five candidate peptides

The MIC values of five candidate peptides (FD4, FD10, FD12, FD14, and FD15), which were selected based on physicochemical properties and antimicrobial effects (determined by the agar punch method), were evaluated using a micro-broth dilution method (Zhou and Zhou, 2018). Serial two-fold dilutions of the candidate peptides were prepared in 96-well plates containing the same microbial inoculum (*E. coli*, *P. aeruginosa* CMCC(B)10104, *B. thuringiensis*, *S. aureus* CMCC(B) 26003, or *C. albicans* CMCC(F) 98001). The plates were incubated (bacteria: 37°C; fungi: 28°C) for 16–18 h. Ampicillin was used as the positive control. The MIC was defined as the lowest peptide concentration where no visible growth occurred (Diao et al., 2014). The experiment was conducted in triplicate.

### 2.8. Predicted helical wheel projection diagrams of six AMPs

Six AMPs (FD10, FD12, FD13, FD14, FD15, and FD16), selected based on antimicrobial activity and the peptide modifications, were processed by Heliquest^5^ (Gautier et al., 2008) in order to generate helical wheel projection diagrams.

### 2.9. Dynamic antimicrobial analysis of three AMPs

During the antimicrobial activity screening it was found that the five antimicrobial AMPs (FD4, FD10, FD12, FD14, and FD15) had inhibitory effects against *E. coli* and *S. aureus* CMCC(B) 26003. Therefore, the follow-up experiments involved these two bacteria, along with the three AMPs with the best antimicrobial effects (FD10, FD12, and FD15). The dynamic antimicrobial effects were analyzed by measuring the effects of the AMPs on bacterial growth over 10 h. First, 100 μL microbial solution (1.0 × 10^6^ CFU/mL) was added to the wells of a 96-well culture plate, and then 100 μL AMP (at 1× and 1/4× MIC) was added and the mixtures were cultured at 37°C (Mishra et al., 2013; Memariani et al., 2016). 100 μL MHB with 100 μL microbial solution was used as the control group. The optical density at 600 nm (OD_600_) was measured every hour for 10 h using a microplate reader (Hou et al., 2007). The experiment was conducted in triplicate.

### 2.10. Time–kill assay of three AMPs

The kill-time curve assay method was used to investigate the bactericidal effects of the essential oil according to the technique described by Joray et al. (2011). A time–kill assay was used to examine the rate at which three AMPs (FD10, FD12, and FD15) killed two microbes (*S. aureus* CMCC(B) 26003 and *E. coli*) *in vitro* over time, based on the number of viable bacteria left at various time points after AMP exposure. Briefly, AMP at 2× MIC was added to MHB containing 10^6^ CFU/mL *S. aureus* CMCC(B) 26003 or *E. coli* and the mixture was then incubated at 37°C and 100 rpm. At various time points, a sample was taken, serially diluted, and plated on MHA for colony counting (Flamm et al., 2019).

### 2.11. Enzyme stability assay of three AMPs

AMP at 2× MIC was incubated with the same volume of 1 mg/mL trypsin solution at 37°C for 1 h, followed by a 5 min 95°C exposure to inactive the trypsin. Thereafter, 100 μL of the resulting solution was added to 96-well plates, followed by 100 μL microbial suspension (1 × 10^6^ CFU/mL). Sterile water with 1 × 10^6^ CFU/mL microbial suspension was used as the blank control. After culturing at 37°C for 16–18 h, colony counting was conducted (Tresnak and Hackel, 2020). The experiment was conducted in triplicate.

### 2.12. Salt sensitivity assays of three AMPs

*E. coli* and *S. aureus* CMCC(B) 26003 (1 × 10^6^ CFU/mL) were incubated in MHB with various final physiological concentrations of serum salts (150 mM NaCl, 4.5 mM KCl, 1 mM MgCl_2_, and 4 mM FeCl_3_). 50 μL of these *E. coli* or *S. aureus* CMCC(B) 26003 suspensions and 50 μL of AMP (serially diluted two-fold to a final concentration of 256 μg/mL) were added to a 96-well plate and incubated at 37°C for 16–18 h (Chou et al., 2016). The MIC values were then determined. The experiment was conducted in triplicate.

### 2.13. Hemolysis and cytotoxicity assays of three AMPs

The hemolytic activity of AMPs on mammalian erythrocytes was determined by optical absorption (Lee et al., 2011). Briefly, fresh mouse erythrocytes were collected, washed, and resuspended in phosphate-buffered saline PBS at a concentration of 8% (*v/v*). Thereafter, 100 μL of this solution was added to a 96-well plate containing an equal volume of AMP (1–256 μg/mL) and incubated at 37°C for 1 h. The positive control was 100 μL 1% Triton X-100 and 100 μL 8% erythrocytes, and the negative control was 100 μL phosphate-buffered saline PBS and 100 μL 8% erythrocytes. Next, each mixture was centrifuged at 1,200 g for 15 min, and 100 μL of the supernatant was transferred to a clean 96-well plate to measure the light absorption value at 576 nm with a microplate reader. The percent hemolysis was calculated using the following formula: *Percent hemolysis* = [(A–A_0_)/(A_+_–A_0_)] × 100%, where A, A_+_, and A_0_ represent the absorbance of the peptide sample and positive and negative controls, respectively (Li et al., 2020).

The cytotoxicity of AMPs toward mouse RAW 264.7 macrophages was determined by MTT assays (Meng and Kumar, 2007). Cells were added into 96-well microtiter plates (2.5 × 10^4^ cells/well) and incubated for 24 h. After another 24 h incubation with Amp and FD10, FD12, FD15 (1 to 256 μg/mL), MTT solution was added and incubated for 4 h. Followed the removing of the MTT, dimethyl sulfoxide (DMSO) (150 μL/well) was added and the absorbance was measured at 570 nm. Untreated cells were used as a control. The cell survival rate was calculated using the following formula: *survival rate* (%) = (Abs_570 nm_ of treated sample/Abs_570 nm_ of control) × 100% (Wang et al., 2018). The cytotoxicity detection was repeated in triplicate.

### 2.14. Statistical analysis

All data are presented as mean ± standard deviation (SD), with “*n*” represents the number of samples. One-way analysis or two-way analysis of variance (ANOVA) in SPSS software was used to analyze significant differences between groups. *p* < 0.05 indicated that the differences were statistically significant. GraphPad Prism 8.0 (GraphPad Software, United States) was used for statistical computing and plotting.

## 3. Results

### 3.1. Template peptides and secondary structure prediction

After comparing the *Protaetia brevitarsis* Lewis genomic sequence to known AMP sequences in a database, nine aligned sequences were identified and used as template peptides (AP02030, AP02257, AP02096, AP00489, AP01575, AP02128, AP01540, AP02012, and AP00208) (Supplementary Figure S1).

PHYRE^2^^6^ was used to predict the secondary structure of the nine template peptides to clearly and intuitively observe their structures, and to help to predict their functions. They all have typical secondary structures, i.e., α-helical structures (green spirals) and β-pleated structures (blue arrows) (other lines are random coil regions) (Supplementary Figure S2). The figure, which indicates the confidence of predictions (decreasing from red to purple), shows that the α-helical region prediction confidence is high (mostly in red). AMPs mainly depend on their α-helical structures, while the β-pleated structures were treated as auxiliary structures in terms of the subsequent related design work.

### 3.2. Screening for candidate peptides

Based on the nine template peptides, key truncated sequences were identified (Supplementary Tables S1–S9), and 16 candidate peptides, designated FD1–16, were designed (Table 1). FD13(ANWDKVIR) contains a negatively charged amino acid residue, which was detrimental to the interaction of the antimicrobial peptide with bacterial membranes, so it could be considered to be replaced with Lys, which becomes FD14 (ANWKKVIR), thereby increasing the net charge from +1 to +3 without changing the hydrophobic amino acid ratio. The polypeptide sequence of FD16 (STLHLVLRRR) was the C-terminal sequence of the template peptide, and the control peptide FD15 (STLHLVLRR-NH_2_) could be set up, and -NH_2_ could be added to the FD16 terminal in order to preserve the integrity of functional activity.

**Table 1:** Amino acid sequences of candidate peptides.

Peptide	Sequence	Peptide	Sequence
FD1	VACAKRVVR	FD9	SLARAGKVR
FD2	LARTLKRLGM	FD10	VFRLKKWIQKVI
FD3	IRAWVAWRNR	FD11	FPVGRVHRLL
FD4	IVHLLTKMTK	FD12	KIYVLLRRQA
FD5	AFQRTIRKFL	FD13	ANWDKVIR
FD6	KLLVPRCR	FD14	ANWKKVIR
FD7	RIGRLVTRAAF	FD15	STLHLVLRLR-NH_2_
FD8	LVTRAAFHGKKV	FD16	STLHLVLRLR

### 3.3. Physicochemical properties of candidate peptides

The physicochemical properties of the 16 candidate peptides were clearly and concisely compared (Table 2). The net charge of each candidate peptide was 2–4, which was favorable for interacting with the negatively charged bacterial membrane. The high ratio of hydrophobic residues indicated easy insertion into the membrane interior to destroy the bacterial cells. The small molecular weight lowered chemical synthesis costs. The instability index was not high except for FD1, FD15, and FD16, which showed that most of the candidate peptides were relatively stable.

**Table 2:** Comparison of physicochemical properties of candidate peptides.

Peptide	MW (Da)^a^	Net charge	pI	Hydrophobic	HRR^b^ (%)	α-spiral proportion (%)	II^c^	LI^d^
FD1	1,001.26	+3	10.86	0.644	66	100	43.93	118.89
FD2	1,158.47	+3	12.01	0.110	50	80	33.11	127.00
FD3	1,327.56	+3	12.30	−0.650	60	60	12.85	88.00
FD4	1,183.52	+2	10.00	0.580	55	100	30.23	146.00
FD5	1,279.55	+3	12.01	−0.140	50	90	9.00	88.00
FD6	984.27	+3	10.86	−0.025	55	100	12.79	133.75
FD7	1,259.52	+3	12.30	0.391	54	100	1.37	115.45
FD8	1,326.61	+3	11.17	0.167	50	67	−20.70	97.50
FD9	957.142	+3	12.01	−0.278	44	100	−9.98	97.78
FD10	1,557.99	+4	11.26	0.283	58	100	−4.98	145.83
FD11	1,193.46	+2	12.00	0.460	50	80	39.03	136.00
FD12	1,259.56	+3	11.00	0.040	50	100	46.31	156.00
FD13	1,001.15	+1	8.79	−0.725	50	75	−25.44	97.50
FD14	1,014.24	+3	11.17	−0.775	50	75	−14.82	97.50
FD15	1,458.73	+2	12.00	−0.083	50	80	51.55	154.17
FD16	1,207.48	+2	12.00	0.570	50	80	47.52	185.00

^a^Molecular weight; ^b^Hydrophobic residue ratio; ^c^Instability index; ^d^Liposolubility index.

### 3.4. Antimicrobial activity of candidate peptides based on agar punch method

The antimicrobial effects of the 16 candidate peptides against five microbes (*E. coli*, *P. aeruginosa* CMCC(B)10104, *B. thuringiensis*, *S. aureus* CMCC(B) 26003, and *C. albicans* CMCC(F) 98001) were compared to the effects of ampicillin (positive control) and sterile water (negative control). The candidate peptides differed in their inhibitory action against the five microbes, based on the size of the inhibition circles formed (Supplementary Figure S3).

Five peptides (FD4, FD10, FD12, FD14, and FD15) generated inhibition circles, with the growth of the five microbes being prevented in these zones (the remaining candidate peptides did not form inhibition circles). FD4 was effective against *E. coli* and *S. aureus* CMCC(B) 26003, with inhibition circle diameters of 10.12 and 9.47 mm, respectively (Table 3). However, they were much smaller than those of ampicillin (29.37 and 19.98 mm), indicating a lower antimicrobial effect. FD10 was more effective (larger inhibition circle diameter) than ampicillin against *P. aeruginosa* CMCC(B)10104, *B. thuringiensis*, and *S. aureus* CMCC(B) 26003, and it was the only candidate peptide with activity against fungi (*C. albicans* CMCC(F) 98001), indicating excellent inhibitory effects. FD12 was comparable to ampicillin against *B. thuringiensis* and *S. aureus* CMCC(B) 26003, indicating antimicrobial activity against Gram-positive bacteria. FD14 and FD15 were both much less effective than ampicillin.

**Table 3:** Diameter of the inhibition zone of 16 peptides.

Peptide	Inhibition circle diameter (mm)				
	*E. coli*	*P. aeruginosa* CMCC(B)10104	*B. thuringiensis*	*S. aureus* CMCC(B) 26003	*C. albicans* CMCC(F) 98001
FD1	0	0	0	0	0
FD2	0	0	0	0	0
FD3	0	0	0	0	0
FD4	10.12 ± 0.26	0	0	9.47 ± 0.11	0
+	29.37 ± 0.31	14.48 ± 0.29	15.97 ± 0.49	19.98 ± 0.42	19.69 ± 0.53
−	0	0	0	0	0
FD5	0	0	0	0	0
FD6	0	0	0	0	0
FD7	0	0	0	0	0
FD8	0	0	0	0	0
+	27.03 ± 0.47	15.10 ± 0.43	17.95 ± 0.36	14.35 ± 0.49	16.16 ± 0.51
−	0	0	0	0	0
FD9	0	0	0	0	0
FD10	21.52 ± 0.32	20.71 ± 0.26	17.19 ± 0.21	14.93 ± 0.54	13.52 ± 0.30
FD11	0	0	0	0	0
FD12	14.10 ± 0.28	0	13.46 ± 0.36	14.71 ± 0.61	0
+	27.84 ± 0.30	16.52 ± 0.55	13.99 ± 0.61	15.58 ± 0.58	17.74 ± 0.47
−	0	0	0	0	0
FD13	0	0	0	0	0
FD14	10.24 ± 0.23	9.50 ± 0.10	8.09 ± 0.12	10.11 ± 0.41	0
FD15	11.18 ± 0.38	0	9.15 ± 0.44	10.02 ± 0.22	0
FD16	0	0	0	0	0
+	30.50 ± 0.21	16.99 ± 0.39	17.47 ± 0.19	16.02 ± 0.34	17.80 ± 0.25
−	0	0	0	0	0

+Is the positive control (Ampicillin). −Is the negative control (sterile water).

### 3.5. Further antimicrobial activity of candidate peptides

Based on the physicochemical properties and antimicrobial effects of the candidate peptides determined using the agar punch method, five (FD4, FD10, FD12, FD14, and FD15) were selected for further antimicrobial activity analysis. FD10 had an antimicrobial effect on all five microbes at a low MIC; the MIC against Gram-negative bacteria *E. coli* and *P. aeruginosa* CMCC(B)10104 was as low as 8 μg/mL, while those against Gram-positive *B. thuringiensis* and *S. aureus* CMCC(B) 26003 were 8 and 16 μg/mL, respectively, and that against *C. albicans* CMCC(F) 98001 was 16 μg/mL. FD12 and FD15 also had antimicrobial activity against *S. aureus* CMCC(B) 26003 with a MIC of 16 μg/mL each and against *E. coli* with a MIC of 32 μg/mL each (Table 4).

**Table 4:** MIC of AMPs.

Species and strains		MIC (μg/mL)				
		FD4	FD10	FD12	FD14	FD15
Gram-negative bacteria	*E. coli*	-	8	32	256	32
	*P. aeruginosa* CMCC(B)10104	-	8	-	256	-
Gram-positive bacteria	*B. thuringiensis*	-	8	128	256	128
	*S. aureus* CMCC(B) 26003	256	16	16	-	16
Fungi	*C. albicans* CMCC(F) 98001	-	16	-	-	-

−Indicated that the MIC was greater than 256 μg/mL.

### 3.6. Helical wheel projection diagrams

Heliquest was used to calculate the helix properties of six AMPs (FD10, FD12, FD13, FD14, FD15, and FD16). Supplementary Figure S4 shows the helical wheel projection diagrams of the AMPs. In this conformation, they did not have different physicochemical properties from those predicted by ExPASy-ProtParam. Both FD10 and FD12 (especially FD10) had a high proportion of electrostatically charged and hydrophobic amino acids, which are favorable for adsorption to the bacterial cell membrane and insertion into the membrane, causing bacterial death. Despite FD14 having a positively charged Lys in place of the negatively charged Asp in FD13, neither FD13 nor FD14 were antimicrobial, with both having a low hydrophilic index. The C-terminal acetylation modification gave FD15 antimicrobial activity. Helical wheel projection diagrams could not be constructed for FD13 and FD14 because the sequences were considered excessively short (only eight amino acid residues).

### 3.7. Dynamic antimicrobial analysis

**Figure 2:**
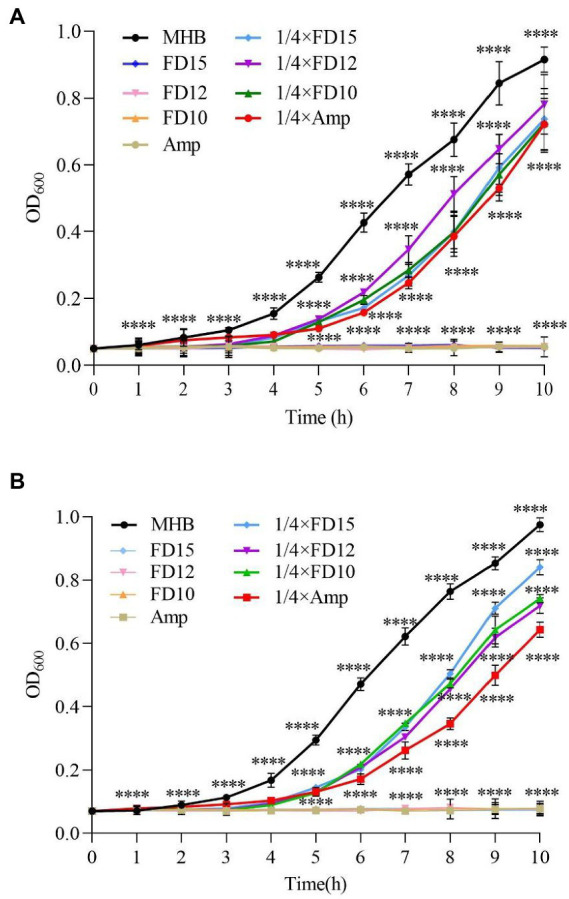
Dynamic antimicrobial analysis of the effects of antimicrobial peptides (AMPs) FD10, FD12, and FD15 against **(A)** *E. coli* (*n* = 3) and **(B)** *S. aureus* CMCC(B) 26003 (*n* = 3). Cells were treated with 1× and 1/4× MIC FD10, FD12, or FD15, with MHB and ampicillin (Amp) as the positive control. The statistical significance of differences was determined using the two-way ANOVA for **(A)** and **(B)**. (*) indicates the significance between Amp, FD10, FD12, FD15 and MHB, ****p < 0.0001.

In the dynamic antimicrobial analysis, the OD_600_ of the microbial solutions were essentially unchanged until 1 h, indicating slow microbe proliferation (Figure 2). At 1× MIC, for both AMPs and ampicillin, *E. coli* and *S. aureus* CMCC(B) 26003 growth was almost inhibited at 10 h (relative to the control [MHB with microbial solution]).

Ampicillin at 1/4× MIC led to slow *E. coli* growth after 1 h and increased growth after 2 h, while the AMPs at 1/4× MIC led to *E. coli* growth only after 3–4 h, and after 4 h, *E. coli* continued to grow. FD10 had a significantly higher inhibitory effect against *E. coli* than the other AMPs (Figure 2A). Similarly, ampicillin at 1/4× MIC led to *S. aureus* CMCC(B) 26003 growth at around 2 h, while AMPs at 1/4× MIC led to slow growth after 3 h (Figure 2B), and after 4 h, the number of *S. aureus* CMCC(B) 26003 began to increase more rapidly.

This indicates that, compared to ampicillin, the AMPs exhibited good microbial inhibition and broad antimicrobial activity even at concentrations below the MIC.

### 3.8. Time–kill assay

**Figure 3:**
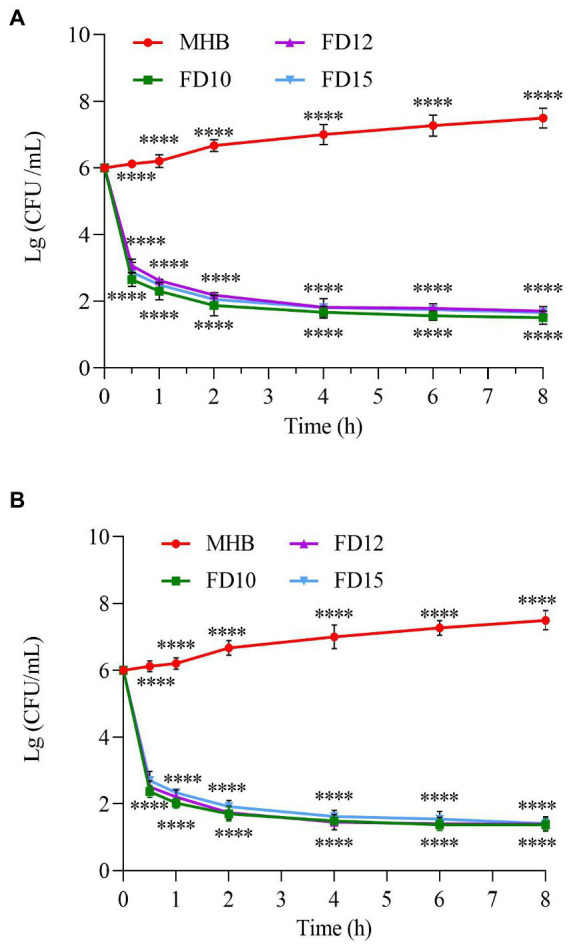
Time–kill curve of **(A)** *E. coli* (*n* = 3) and **(B)** *S. aureus* CMCC(B) 26003 (*n* = 3). Cells were treated with 2× MIC of FD10, FD12, or FD15, with MHB as the control. ****p < 0.0001 based on two-way ANOVA.

All three tested AMPs (FD10, FD12, and FD15) were bactericidal immediately after *E. coli* was exposed to them, with FD10 having the steepest curve and the fastest decline at any given time point, indicating that it was the most effective at killing *E. coli*, followed by FD12 and then FD15 (Figure 3A). The bactericidal effect of the three AMPs on both bacteria (*E. coli* and *S. aureus* CMCC(B) 26003) was most rapid before 0.5 h, with a large reduction in the number of colonies, and the rate of bactericidal action gradually decreased with time until the curves plateaued. Similarly, all three AMPs were bactericidal immediately after *S. aureus* CMCC(B) 26003 was exposed to them, with FD10 and FD12 causing a more rapid decline in *S. aureus* CMCC(B) 26003 than FD15, indicating that they were better than FD15 at killing *S. aureus* CMCC(B) 26003 (Figure 3B). By 10 h, the inhibitory effect of all three AMPs on *S. aureus* CMCC(B) 26003 decreased and the number of colonies began to increase, but the bacteria grew more slowly in the FD10 group.

### 3.9. Stability of AMPs against trypsin

**Figure 4:**
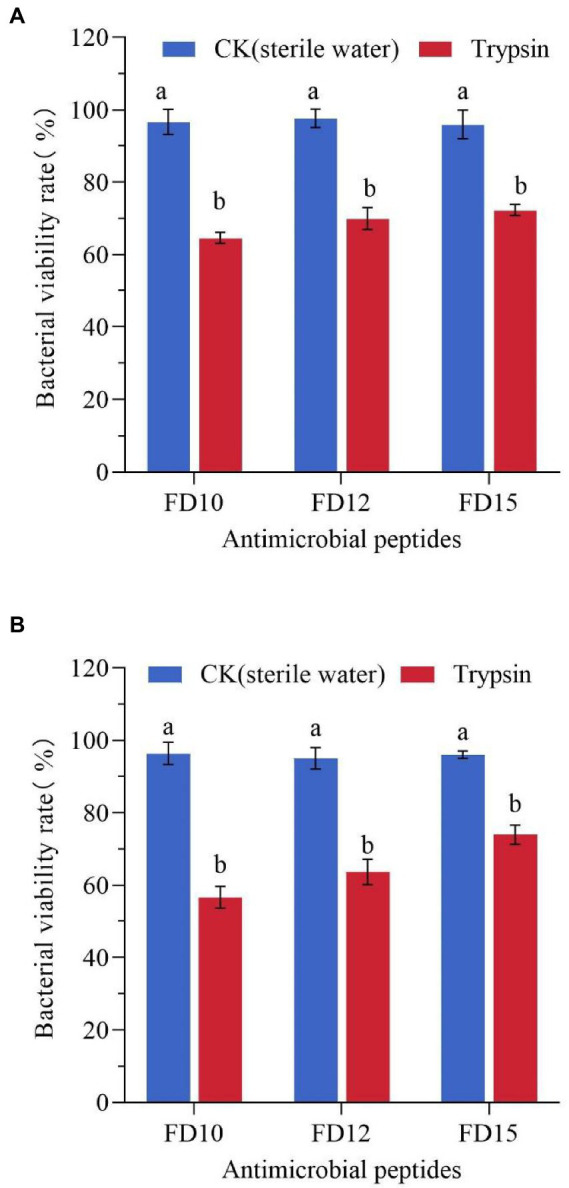
Changes in the antimicrobial activity of AMPs against **(A)** *E. coli* (*n* = 3) and **(B)** *S. aureus* CMCC(B) 26003 (*n* = 3) after trypsin treatment. *E. coli* or *S. aureus* CMCC(B) 26003 were treated with 2× MIC FD10, FD12, or FD15 and the same volume of 1 mg/mL trypsin or sterile water (control). Different lowercase letters (a, b) indicate a significant difference (*p* < 0.05) based on one-way ANOVA.

After being treated with trypsin for 1 h, the antimicrobial activity of the three AMPs was greatly reduced. The antimicrobial activity was less than half of the original activity, with >60% of the bacteria surviving (Figure 4). This shows that the three AMPs were easily decomposed by trypsin and exhibited reduced activity.

### 3.10. Salt sensitivity

The salt sensitivity of antimicrobial agents is generally tested by the addition of physiological concentrations of various salts (Table 5). The antimicrobial activities of the three AMPs (FD10, FD12, and FD15) against *E. coli* and *S. aureus* CMCC(B) 26003 were minimally affected, or even promoted, by the presence of certain monovalent (K^+^) and trivalent (Fe^3+^) cations, while they were slightly decreased in the presence of Na^+^ or Mg^2+^. Among FD10, FD12, and FD15, FD10 was the most stable in the presence of salt; when certain salt ions were present, it maintained or increased its antimicrobial activity.

**Table 5:** MIC of peptides in the presence of physiological salts.

Peptide	Control (mM)^a^	NaCl (mM)^a^	KCl (mM)^a^	MgCl_2_ (mM)^a^	FeCl_3_ (mM)^a^
Gram-negative bacteria *E. coli*					
FD10	8	16	4	16	4
FD12	32	64	16	64	32
FD15	32	64	64	64	16
Gram-positive bacteria *S. aureus* CMCC(B) 26003					
FD10	16	32	8	32	16
FD12	16	32	8	32	32
FD15	16	64	32	64	32

^a^The final concentrations of NaCl, KCl, MgCl_2_, and FeCl_3_ were 150 mM, 4.5 mM, l mM, and 4 mM, respectively, and the control MIC values were determined in the absence of these physiological salts.

### 3.11. Hemolytic effects and cytotoxicity of AMPs

**Figure 5:**
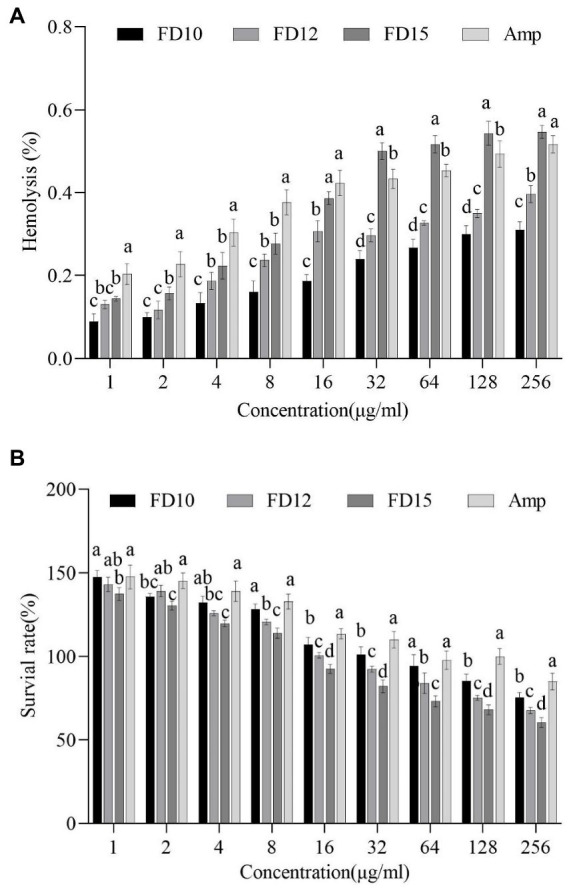
**(A)** Hemolytic activity against mouse erythrocytes (*n* = 3) and **(B)** cytotoxicity against mouse RAW 264.7 macrophages (*n* = 3) of FD10, FD12, FD15, and ampicillin (Amp). Different lowercase letters (a, b, c, d) indicate a significant difference (*p* < 0.05) within each identical-concentration experiment, based on one-way ANOVA. ‘a’ indicates the largest mean in the group; ‘b’, ‘c’, and ‘d’ indicate the largest, second largest, and third largest mean, respectively, that is significantly different from ‘a’.

The percentage of mouse erythrocyte hemolysis after FD10 and FD12 treatment (at 256 μg/mL) were 0.31 and 0.40%, respectively, which were lower than that of ampicillin (0.52%), while the rate after FD15 treatment was 0.55%, which was higher than that of ampicillin (Figure 5A). The survival rates of mouse RAW 264.7 macrophages after FD10, FD12, and FD15 treatment (at 256 μg/mL) were 75.37, 67.56, and 60.50%, respectively, which were lower than that of ampicillin (84.90%) (Figure 5B). The results showed that the hemolytic effects and cytotoxicity of FD10 and FD12 were lower than those of ampicillin, while the hemolytic effect of FD15 was higher than that of ampicillin.

## 4. Discussion

With the overuse of traditional antibiotics for animal and human health, a large number of super-resistant bacteria have emerged, which seriously threatens animal and human health and the development of industries such as the aquaculture industry. Therefore, the exploration and development of new antimicrobial drugs have become a hot research area. Due to the broad-spectrum antimicrobial properties of AMPs and their unique antimicrobial mechanisms, it is difficult for bacteria to become resistant to AMPs, and AMPs are one of the most potent agents to replace antibiotics. At present, there are many studies on insect AMPs, but few studies on the AMPs of *P. brevitarsis* Lewis larvae.

In this study, nine natural AMP sequences were used as templates to design small-molecule AMPs with strong antimicrobial activity and low cytotoxicity. Traditionally, the secondary structures of AMPs mainly include α-helix, β-pleated, random coil, and loop structures. The antimicrobial activity of AMPs is associated with their structures (Hilpert et al., 2006). Therefore, the template peptide sequences with secondary structures were retained. In addition, while most natural AMPs are long, truncated peptides have been shown to retain antimicrobial activity (Wang et al., 2022). By generating various heterogeneous peptides by truncating AMP sequences at the N- or C-terminus of the peptide chain, researchers have demonstrated experimentally that retaining only 10 amino acid residues at the N-terminus of AMPs still results in bacteriostatic and neutrophilic activity (Kwon et al., 2014). In the current study, we designed truncated peptides using the natural AMPs as templates along with bioinformatics software. However, N- and C-terminal amino acids were not removed, replaced, or added. FD15 (STLHLVLRLR-NH_2_) had an obvious antimicrobial effect but FD16 (STLHLVLRLR) had no effect without -NH_2_. Although C-terminal acetylation is not a requirement for antimicrobial activity, it can increase the charge of peptides and enhance their interaction with microbial membranes. In contrast, adding amino acid residues or truncating sequences at the N-terminus makes it more difficult for the AMP to insert into the membrane, which reduces the AMP’s activity (Cerovsky et al., 2011). C-terminal acetylation provides additional hydrogen bonds to the α-helix and there has been reported to be a significant correlation between this and biological activity (Rangel et al., 2011; Dos Santos Cabrera et al., 2019). Moreover, cationic AMPs can bind to the bacterial cell membrane, which is negatively charged. When the net charge of the truncated peptide is low (<2), Arg or Lys (with a higher isoelectric point) can be used to replace Glu and Asp to increase the net charge of AMPs (Arias et al., 2018). Due to containing a negatively charged amino acid residue, FD13 (ANWDKVIR) could not readily interact with bacterial membranes. Based on FD13, FD14 (ANWKKVIR) was designed by replacing Asp with Lys. However, neither had antimicrobial activity, potentially due to their weak hydrophilicity. On the other hand, when the hydrophobicity of the AMPs is low (<40%), hydrophobic amino acid residues can be added. The helical wheel projection diagrams predicted the hydrophobic distance of individual peptides. The hydrophobic moments (μH) of peptides can be utilized to represent their amphiphilic nature (Zhao and London, 2006). Peptide-membrane interactions are strongly affected by using the amphiphilic structure of peptides. The amphiphilic property of peptides permits amino acids to create hydrophobic moments on the facets of the helix (Tan et al., 2022).

An important factor for identifying candidate AMPs is the MIC. In general, the MIC should be <16 μg/mL to preliminarily qualify (Barreto-Santamaria et al., 2019). In this study, FD10 (VFRLKKWIQKVI) was shown to have antimicrobial activity against both bacteria and a fungus at low MIC values of 8–16 μg/mL. More importantly, FD10 had stronger antimicrobial effects than ampicillin in some respects, indicating excellent antimicrobial function. Additionally, FD12 and FD15 had antimicrobial activity against *S. aureus* CMCC(B) 26003, which is resistant to antibiotics (Chambers and DeLeo, 2009). Moreover, although most natural AMPs cannot be widely used because of their high cytotoxicity and hemolytic effects (Kubicek-Sutherland et al., 2017), FD10, FD12, and FD15 are promising antimicrobial agents because of their strong activity and low cytotoxicity and hemolytic effects. The inactivity of other AMPs may be related to their structural stability or hydrophilicity (Diao et al., 2014; Li et al., 2019). The dynamic antimicrobial analysis showed that the AMPs could completely kill bacteria and act rapidly at 1× MIC, and the microbes would also be affected at 1/4× MIC, indicating that FD10, FD12, and FD15 had rapid and extensive antimicrobial activity. According to the time–kill curves, FD10, FD12, and FD15 had obvious antimicrobial effects, with highly rapidly killing *E. coli* and *S. aureus* CMCC(B) 26003 before 0.5 h in the colony numbers. The best antimicrobial activity was observed for FD10. The designed peptides exert a similar rapid antimicrobial activity as what has been observed for naturally produced antimicrobial peptides. This rapid activity has been linked to the lower resistance development potential against AMPs (Lazzaro et al., 2020; Gan et al., 2021; Sarkar et al., 2021; Vanzolini et al., 2022).

However, the application of AMPs is limited due to the high cost of synthesis and the difficulties regarding extraction and purification. At present, two methods are widely used: the genetic engineering method and the chemical synthesis method (Wang et al., 2022). The former involves obtaining AMPs by constructing an expression system. However, there are several problems: AMPs cannot be easily detected because of their small molecular weight; their isolation and purification are complicated; and the spatial structure may not be consistent with the designed peptides. Therefore, the chemical synthesis method is widely used to synthesize AMPs because the isolation and purification procedures are simple, and the resulting AMPs are highly pure (Chen et al., 2022). Moreover, the antimicrobial mechanism of the AMPs in this study is still not clear. Further work should focus on the impacts of AMPs on the cell membrane and probing the potential intracellular targets. The mechanisms could be explored by observing the structural changes of cell membranes using transmission and scanning electron microscopy.

Furthermore, some studies have shown that AMPs are not stable (Lai et al., 2022). Thus, salt stability and enzyme stability experiments were carried out and the results showed that FD10 had the best stability and was more stable than FD12 and FD15. In addition, it was previously shown that AMPs can be coupled with functional polymers to form AMP–polymer conjugates and the antimicrobial effect increased 10-fold compared to the original AMP, with a largely unchanged hemolysis index (Liu et al., 2006). This should be researched further in future. Moreover, a peptide variant designed to be cationic exhibited stronger antimicrobial activity than before the modification (Li et al., 2020). Based on these findings, more novel AMPs with strong antimicrobial activity are expected to be developed.

There are several limitations in this study. First, only mouse cells were used to verify the cytotoxicity and hemolytic effects of AMPs. As the AMPs were developed to use to improve human health by enriching the types of AMPs available, erythrocytes from humans or other species could be selected to verify the cytotoxicity and hemolytic effects of the AMPs (Houyvet et al., 2018; Chowdhury et al., 2020). Second, only ampicillin was used as the positive control, which made our experimental results conservative. There are many classifications of antibiotics, such as peptides (vancomycin), β-lactams (ampicillin), and quinolones (norfloxacin), and as the antimicrobials in this study were polypeptides, the selected control group antibiotics are preferably peptide antibiotics (Yang et al., 2018), which should be explored in future studies. Our study preliminarily verified the physiological activity of the screened AMPs, in follow-up experiments, we should study the mechanism of action of the AMPs.

In summary, 16 small-molecule potential AMPs were designed in this study. Among them, FD10 had the best antimicrobial activity, especially regarding antifungal activity. FD12 and FD15 also exhibited activity against *S. aureus*. Some of the small-molecule AMPs delayed the growth of microorganisms better than ampicillin. Time–kill assays showed that FD10, FD12, and FD15 act rapidly and can inhibit the growth of *E. coli* and *S. aureus* CMCC(B) 26003 in a short time. The findings indicate that FD10 in particular may be a novel promising antimicrobial agent. This study provides a basis for the design of small-molecule AMPs and enriches AMP resources.

## Data availability statement

The original contributions presented in the study are included in the article/Supplementary material, further inquiries can be directed to the corresponding authors.

## Ethics statement

The experiments were approved by the Institutional Animal Care and Use Committee of the Northeast Agricultural University (SRM-11) in 2022 (China).

## Author contributions

QF, DC, RL, and CS contributed to the conception and design of the study. QF and JS organized the database. QF performed the statistical analysis and wrote the first draft of the manuscript. DC and XL wrote sections of the manuscript. DC, RL, and HL reviewed and edited the manuscript. QF and DC contributed equally to this work. All authors contributed to the manuscript revision and read and approved the submitted version of the manuscript.

## Funding

This research was funded by Heilongjiang Provincial National Science Foundation (LH2020C007), China from 2020-07-01 to 2023-07-01.

## Conflict of interest

The authors declare that the research was conducted in the absence of any commercial or financial relationships that could be construed as a potential conflict of interest.

## Publisher’s note

All claims expressed in this article are solely those of the authors and do not necessarily represent those of their affiliated organizations, or those of the publisher, the editors and the reviewers. Any product that may be evaluated in this article, or claim that may be made by its manufacturer, is not guaranteed or endorsed by the publisher.

## Footnotes

^1^http://aps.unmc.edu/AP/

^2^http://www.camp.bicnirrh.res.in/

^3^http://aps.unmc.edu/AP/prediction/prediction_main.php

^4^http://web.expasy.org/protparam/

^5^https://heliquest.ipmc.cnrs.fr/

^6^http://www.sbg.bio.ic.ac.uk/phyre2/htmL/
