# Supplementary material for: Prediction and bioactivity of small-molecule antimicrobial peptides from Protaetia brevitarsis Lewis larvae
Source: Front Microbiol. 2023 Mar 16;14:1124672. doi: 10.3389/fmicb.2023.1124672 (PMC10060639; doi:10.3389/fmicb.2023.1124672)
Supplement: Supplementary file 1 [file Data_Sheet_1.docx]

Prediction and Bioactivity of Small-Molecule Antimicrobial Peptides from *Protaetia brevitarsis* Lewis larvae

Qian Fu^1^^,^ ^†^, Dengtian Cao^1, †^, Jing Sun^1^, Xinbo Liu^1^, Haitao Li^1^, Changlong Shu^2, *^, Rongmei Liu^1, *^

^1^ College of Life Sciences, Northeast Agricultural University, Harbin 150030, China

^2^ State Key Laboratory for Biology of Plant Diseases and Insect Pests, Institute of Plant Protection, Chinese Academy of Agricultural Sciences, Beijing, China

^†^ These authors contributed equally to this work

*** Correspondence:**Changlong Shu
shuchanglong@caas.cn

Rongmei Liu

liurongmei@neau.edu.cn

Supplementary Material

## Supplementary Tables

**Table S1.** Truncated sequence of template peptide AP02030

| **Num.** | **Sequence** | **location** | **MW (Da) ^a^** | **Net charge** | **Haar**  **(%) ^b^** | **Ss ^c^** | **P ^d^** | **Yes/No ^e^** |
| --- | --- | --- | --- | --- | --- | --- | --- | --- |
| 1 | STLHLVLRLR | 65-74 | 1207.48 | +2 | 50 | β-turn | 0.332 | Yes |
| 2 | MQIFVKTLTGKTITLEV | 1-17 | 1922.35 | +1 | 47 | β-turn | 0.164 | Yes |
| 3 | PDQQRLIFAGKQ | 38-49 | 1400.6 | +1 | 33 | β-turn | 0.228 | No |
| 4 | IENVKAKIQDK | 23-33 | 1285.49 | +1 | 36 | α-helix | 0.280 | No |

^a^ Molecular weight (Da). ^b^ Hydrophobic amino acid ratio. ^c^ Secondary structure. ^d^ CAMP predicts AMP possibility. ^e^ APD3 predicts whether it can be AMP.

**Table S2.** Truncated sequence of template peptide AP02257

| **Num.** | **Sequence** | **location** | **MW (Da) ^a^** | **Net charge** | **Haar**  **(%) ^b^** | **Ss ^c^** | **P ^d^** | **Yes/No ^e^** |
| --- | --- | --- | --- | --- | --- | --- | --- | --- |
| 1 | RCELARTLKRL | 5-15 | 1358.674 | +3 | 45 | α-helix | 0.347 | Yes |
| 2 | LARTLKRLGM | 8-17 | 1158.476 | +3 | 50 | α-helix | 0.431 | Yes |
| 3 | IVHLLTKMTK | 13-22 | 1183.518 | +2 | 55 | α-helix | 0.271 | Yes |
| 4 | SLANWMCLAKW | 24-34 | 1338.809 | +1 | 45 | α-helix | 0.400 | Yes |
| 5 | VACAKRVVR | 93-101 | 1001.263 | +3 | 66 | α-helix | 0.572 | Yes |
| 6 | IRAWVAWRNR | 106-115 | 1327.56 | +3 | 60 | α-helix | 0.442 | P or W |

^a^ Molecular weight (Da). ^b^ Hydrophobic amino acid ratio. ^c^ Secondary structure. ^d^ CAMP predicts AMP possibility. ^e^ APD3 predicts whether it can be AMP. P or W represents antimicrobial peptides that may be Pro or Trp classes.

**Table S3.** Truncated sequence of template peptide AP02096

| **Num.** | **Sequence** | **location** | **MW (Da) ^a^** | **Net charge** | **Haar**  **(%) ^b^** | **Ss ^c^** | **P ^d^** | **Yes/No ^e^** |
| --- | --- | --- | --- | --- | --- | --- | --- | --- |
| 1 | SLARAGKVR | 5-13 | 957.142 | +3 | 44 | α-helix | 0.505 | Yes |
| 2 | YNRRFVNVV | 38-46 | 1166.351 | +2 | 44 | α-helix | 0.251 | Yes |
| 3 | TGRAKRRMQ | 29-37 | 1103.315 | +4 | 22 | α-helix | 0.497 | No |

^a^ Molecular weight (Da). ^b^ Hydrophobic amino acid ratio. ^c^ Secondary structure. ^d^ CAMP predicts AMP possibility. ^e^ APD3 predicts whether it can be AMP.

**Table S4.** Truncated sequence of template peptide AP00489

| **Num.** | **Sequence** | **location** | **MW (Da) ^a^** | **Net charge** | **Haar**  **(%) ^b^** | **Ss ^c^** | **P ^d^** | **Yes/No ^e^** |
| --- | --- | --- | --- | --- | --- | --- | --- | --- |
| 1 | RAGLQFPVG | 20-28 | 944.104 | +1 | 44 | α-helix  β-pleated | 0.304 | No |
| 2 | FPVGRVHRLL | 25-34 | 1193.464 | +2 | 50 | α-helix  β-pleated | 0.466 | Yes |

^a^ Molecular weight (Da). ^b^ Hydrophobic amino acid ratio. ^c^ Secondary structure. ^d^ CAMP predicts AMP possibility. ^e^ APD3 predicts whether it can be AMP.

**Table S5.** Truncated sequence of template peptide AP01575

| **Num.** | **Sequence** | **location** | **MW (Da) ^a^** | **Net charge** | **Haar**  **(%) ^b^** | **Ss ^c^** | **P ^d^** | **Yes/No ^e^** |
| --- | --- | --- | --- | --- | --- | --- | --- | --- |
| 1 | IVERPVCKDSTR | 4-15 | 1402.637 | +1 | 33 | α-helix  β-turn | 0.201 | No |
| 2 | RWYQMGIVS | 55-63 | 1139.345 | +1 | 44 | β-turn | 0.228 | Yes |
| 3 | VFRLKKWIQKVI | 80-91 | 1557.98 | +4 | 58 | α-helix | 0.981 | Yes |

^a^ Molecular weight (Da). ^b^ Hydrophobic amino acid ratio. ^c^ Secondary structure. ^d^ CAMP predicts AMP possibility. ^e^ APD3 predicts whether it can be AMP.

**Table S6.** Truncated sequence of template peptide AP02128

| **Num.** | **Sequence** | **location** | **MW (Da) ^a^** | **Net charge** | **Haar**  **(%) ^b^** | **Ss ^c^** | **P ^d^** | **Yes/No ^e^** |
| --- | --- | --- | --- | --- | --- | --- | --- | --- |
| 1 | ANWDKVIR | 51-58 | 1001.15 | +1 | 50 | α-helix  β-pleated | 0.225 | Yes |
| 2 | YSIHGSWRW | 67-75 | 1191.34 | +1 | 33 | α-helix | 0.321 | No |

^a^ Molecular weight (Da). ^b^ Hydrophobic amino acid ratio. ^c^ Secondary structure. ^d^ CAMP predicts AMP possibility. ^e^ APD3 predicts whether it can be AMP.

**Table S7.** Truncated sequence of template peptide AP01540

| **Num.** | **Sequence** | **location** | **MW (Da) ^a^** | **Net charge** | **Haar**  **(%)^b^** | **Ss^c^** | **P ^d^** | **Yes/No ^e^** |
| --- | --- | --- | --- | --- | --- | --- | --- | --- |
| 1 | IVHLLTKMTK | 13-22 | 1183.518 | +2 | 50 | α-helix | 0.478 | Yes |
| 2 | AFQRTIRKFL | 25-34 | 1279.55 | +3 | 50 | α-helix | 0.188 | Yes |
| 3 | KLLVPRCR | 44-51 | 984.276 | +3 | 55 | α-helix | 0.554 | P or W |
| 4 | KPKAICSHV | 70-78 | 982.208 | +2 | 44 | α-helix | 0.323 | No |

^a^ Molecular weight (Da). ^b^ Hydrophobic amino acid ratio. ^c^ Secondary structure. ^d^ CAMP predicts AMP possibility. ^e^ APD3 predicts whether it can be AMP. P or W represents antimicrobial peptides that may be Pro or Trp classes.

**Table S8.** Truncated sequence of template peptide AP02012

| **Num.** | **Sequence** | **location** | **MW (Da) ^a^** | **Net charge** | **Haar**  **(%) ^b^** | **Ss ^c^** | **P ^d^** | **Yes/No ^e^** |
| --- | --- | --- | --- | --- | --- | --- | --- | --- |
| 1 | VKVGINGFGRI | 1-11 | 1159.396 | +2 | 45 | β-turn | 0.372 | P or W |
| 2 | RIGRLVTRAAF | 10-20 | 1259.524 | +3 | 54 | α-helix | 0.372 | P or W |
| 3 | LVTRAAFHGKKV | 14-25 | 1326.602 | +3 | 50 | α-helix | 0.431 | Yes |
| 4 | KKVEVVAIN | 23-31 | 999.213 | +1 | 55 | α-helix | 0.281 | Yes |

^a^ Molecular weight (Da). ^b^ Hydrophobic amino acid ratio. ^c^ Secondary structure. ^d^ CAMP predicts AMP possibility. ^e^ APD3 predicts whether it can be AMP. P or W represents antimicrobial peptides that may be Pro or Trp classes.

**Table S9.** Truncated sequence of template peptide AP00208

| **Num.** | **Sequence** | **location** | **MW (Da) ^a^** | **Net charge** | **Haar**  **(%) ^b^** | **Ss ^c^** | **P ^d^** | | **Yes/No ^e^** |
| --- | --- | --- | --- | --- | --- | --- | --- | --- | --- |
| 1 | KDWIKEKIYV | 12-20 | 1321.572 | +1 | 40 | α-helix | 0.425 | No | |
| 2 | KIYVLLRRQA | 18-27 | 1259.56 | +3 | 50 | α-helix | 0.469 | Yes | |

^a^ Molecular weight (Da). ^b^ Hydrophobic amino acid ratio. ^c^ Secondary structure. ^d^ CAMP predicts AMP possibility. ^e^ APD3 predicts whether it can be AMP.

## Supplementary Figures

| 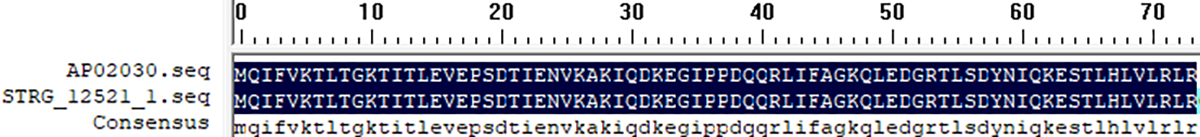  **Figure S1A.** Antimicrobial peptide AP02030 was STRG_12521_1 compared with the *Protaetia brevitarsis* Lewis larvae. |
| --- |
| 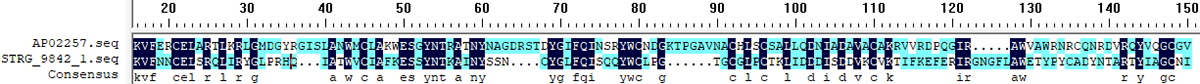 |
| **Figure S1B.** Antimicrobial peptide AP02257 was STRG_9842_1 compared with the gene of the *Protaetia brevitarsis* Lewis larvae. |
| 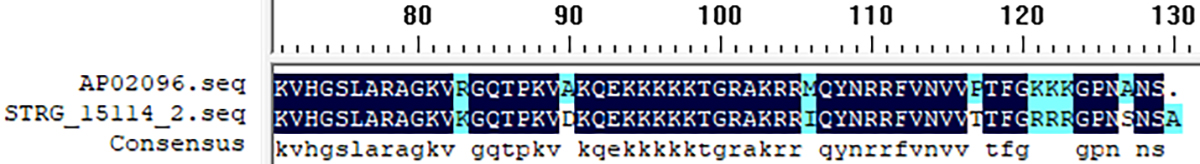 |
| **Figure S1C.** Antimicrobial peptide AP02096 was STRG_15114_2 compared with the gene of the *Protaetia brevitarsis* Lewis larvae. |
| 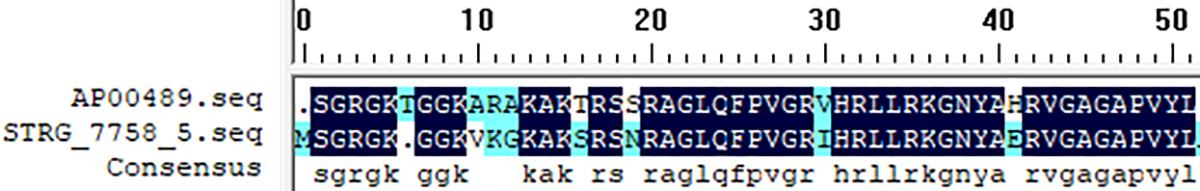 |
| **Figure S1D.** Antimicrobial peptide AP00489 was STRG_7758_5 compared with the *Protaetia brevitarsis* Lewis larvae. |
| 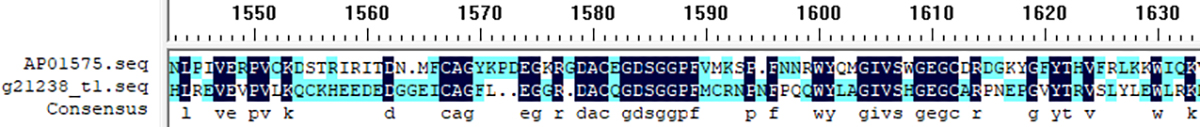 |
| **Figure S1E.** Antimicrobial peptide AP01575 was g21238_t1 compared with the *Protaetia brevitarsis* Lewis larvae. |
| 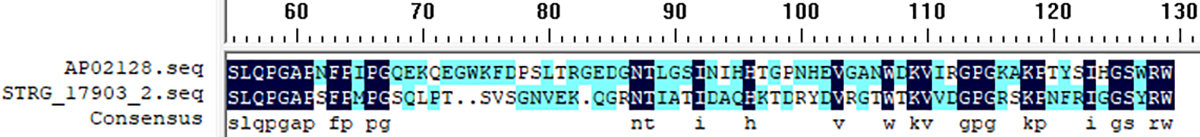 |
| **Figure S1F.** Antimicrobial peptide AP02128 was STRG_17903_2 compared with the *Protaetia brevitarsis* Lewis larvae. |
|  |
| 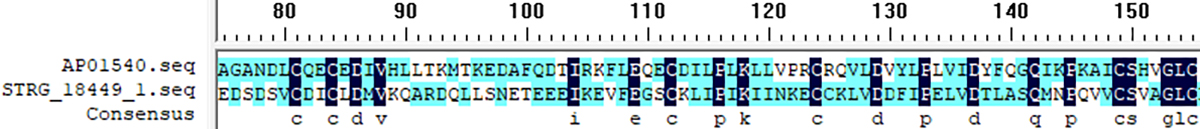 |
| **Figure S1G.** Antimicrobial peptide AP01540 was STRG_18449_1 compared with the *Protaetia brevitarsis* Lewis larvae. |
| 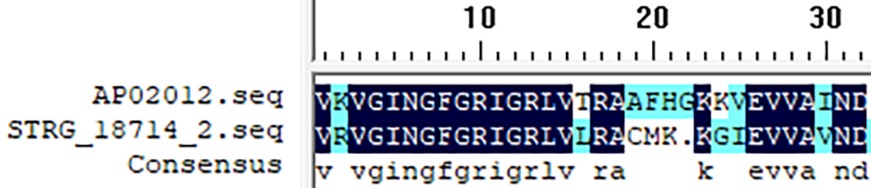 |
| **Figure S1H.** Antimicrobial peptide AP02012 was STRG_18714_2 compared with the gene of the *Protaetia brevitarsis* Lewis larvae. |
| 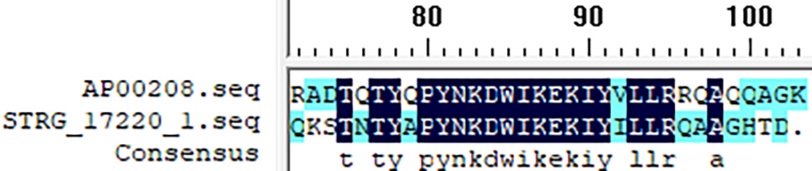 |
| **Figure S1I.** Antimicrobial peptide AP00208 was STRG_17220_1 compared with the *Protaetia brevitarsis* Lewis larvae. |

| 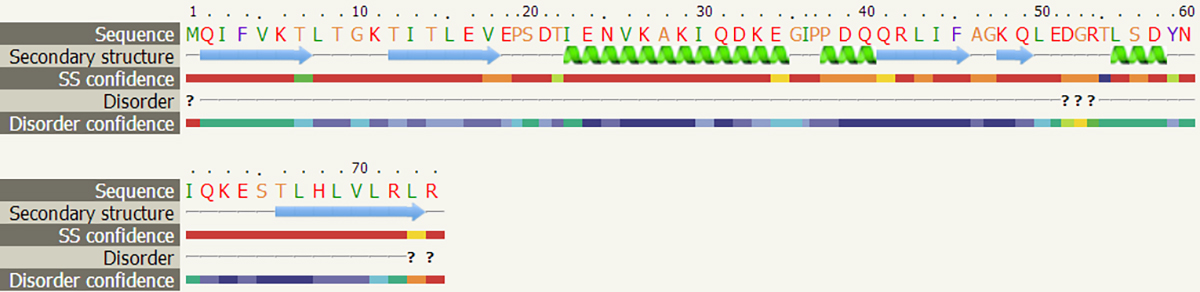  **Figure S2A.** Secondary structure prediction for AP02030. Green: α-helix; Blue: β-pleated; Other straight lines: random coil. |
| --- |
| 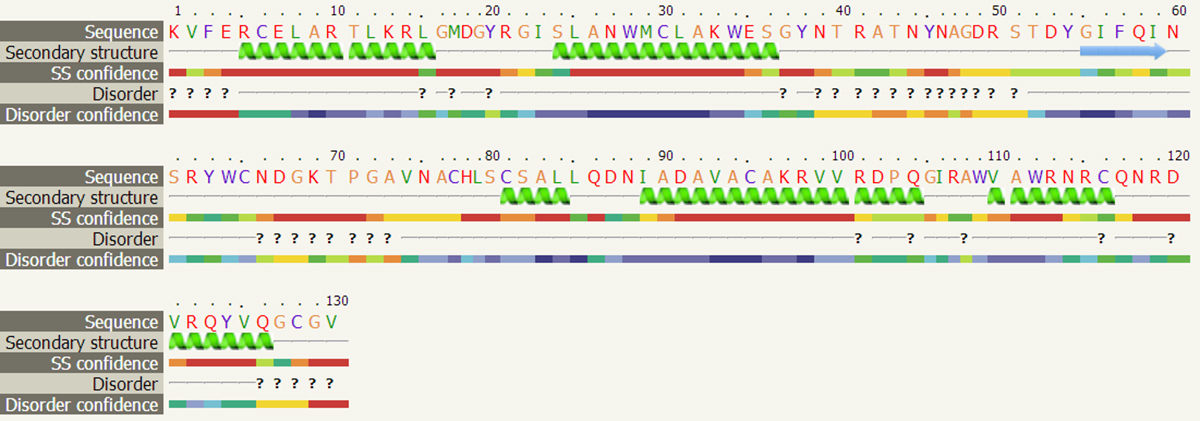 |
| **Figure S2B.** Secondary structure prediction for AP02257. Green: α-helix; Blue: β-pleated; Other straight lines: random coil. |
| 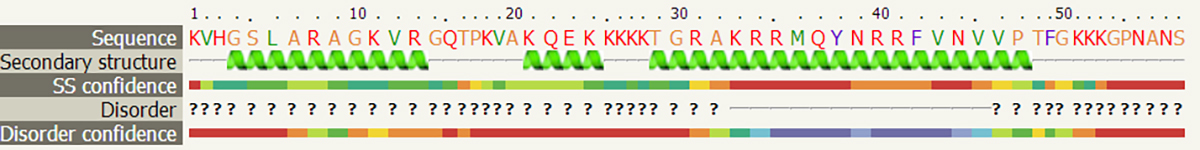 |
| **Figure S2C.** Secondary structure prediction for AP02096. Green: α-helix; Blue: β-pleated; Other straight lines: random coil. |
| 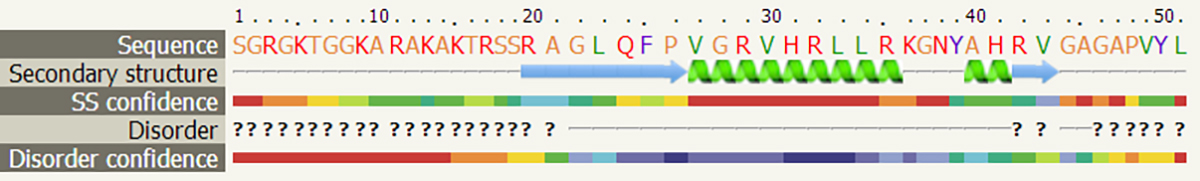 |
| **Figure S2D.** Secondary structure prediction for AP00489. Green: α-helix; Blue: β-pleated; Other straight lines: random coil. |
| 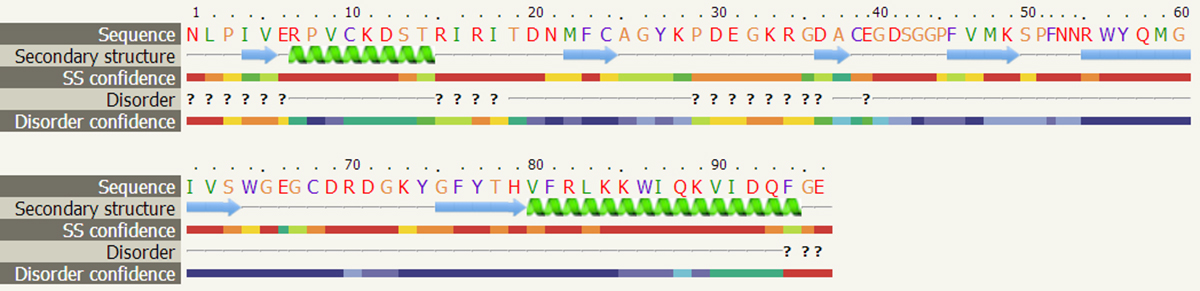 |
| **Figure S2E.** Secondary structure prediction for AP01575. Green: α-helix; Blue: β-pleated; Other straight lines: random coil. |
| 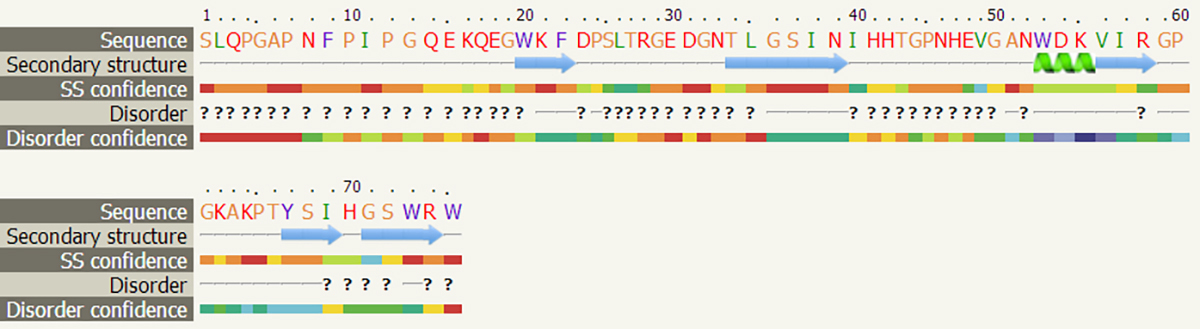 |
| **Figure S2F.** Secondary structure prediction for AP02128. Green: α-helix; Blue: β-pleated; Other straight lines: random coil. |
| 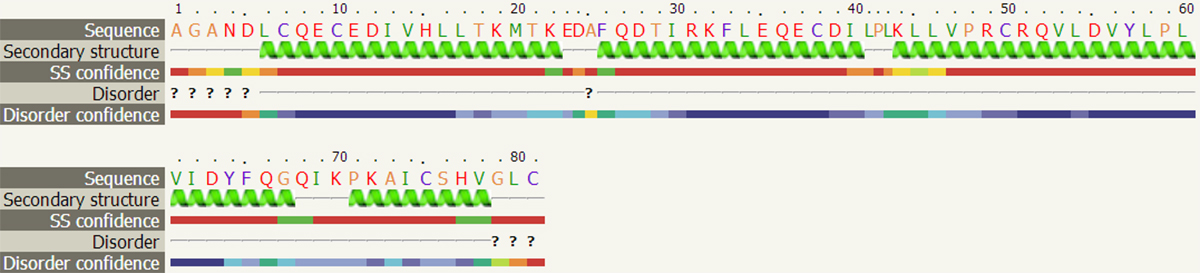 |
| **Figure S2G.** Secondary structure prediction for AP01540. Green: α-helix; Blue: β-pleated; Other straight lines: random coil. |
| 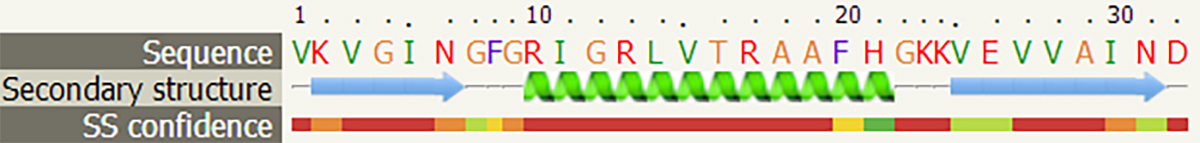 |
| **Figure S2H.** Secondary structure prediction for AP02012. Green: α-helix; Blue: β-pleated; Other straight lines: random coil. |
| 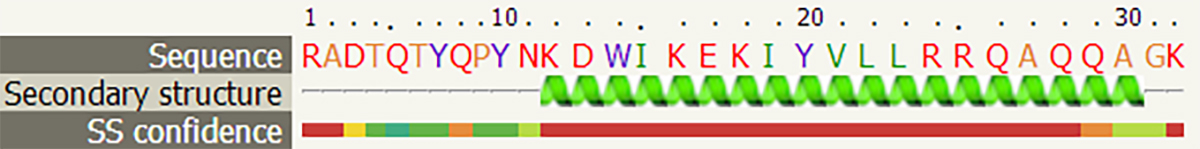 |
| **Figure S2I.** Secondary structure prediction for AP00208. Green: α-helix; Blue: β-pleated; Other straight lines: random coil. |

| 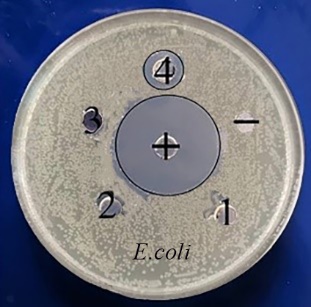 | 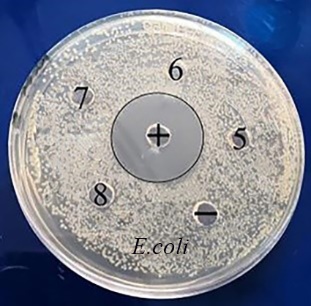 | 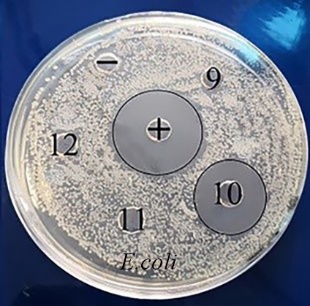 | 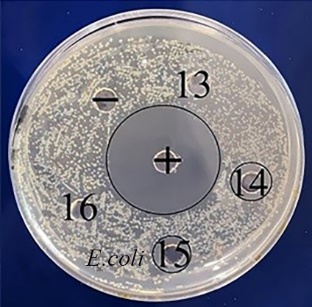 |  |
| --- | --- | --- | --- | --- |
| **Figure S3A.** The antibacterial effect of peptides FD1-FD16 on *E. coli*. The diameter of the Petri dish is 85 mm. The concentration of positive control ampicillin and candidate peptide was 1 mg/mL. *E. coli* at 37°C, cultured for 16-18 h. | | | | |
| 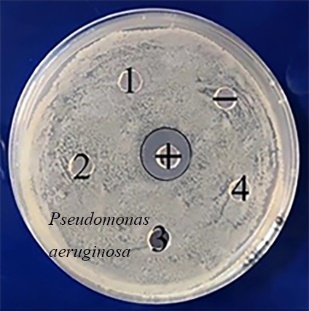 | 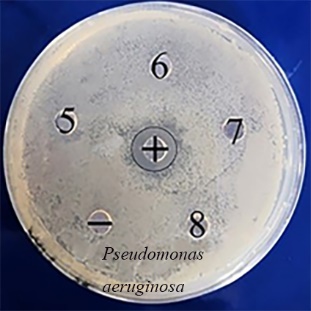 | 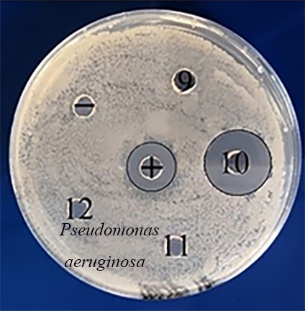 | 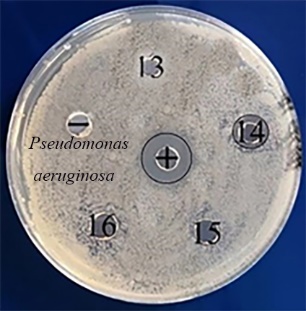 |  |
| **Figure S3B.** The antibacterial effect of peptides FD1-FD16 on *P. aeruginosa* CMCC(B)10104. The diameter of the Petri dish is 85 mm. The concentration of positive control ampicillin and candidate peptide was 1 mg/mL. *P. aeruginosa* CMCC(B)10104 at 37°C, cultured for 16-18 h. | | | | |
| 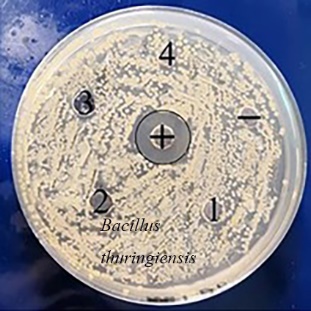 | 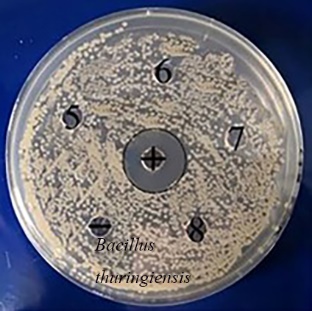 | 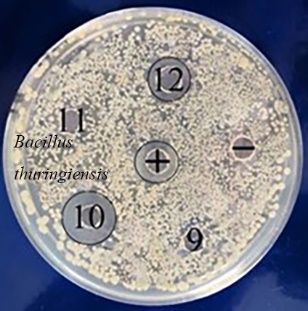 | 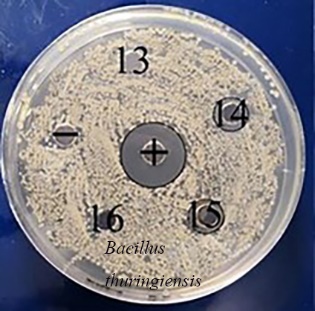 |  |
| **Figure S3C.** The antibacterial effect of peptides FD1-FD16 on *B. thuringiensis*. The diameter of the Petri dish is 85 mm. The concentration of positive control ampicillin and candidate peptide was 1 mg/mL. *B. thuringiensis* at 30°C, cultured for 16-18 h. | | | | |
| 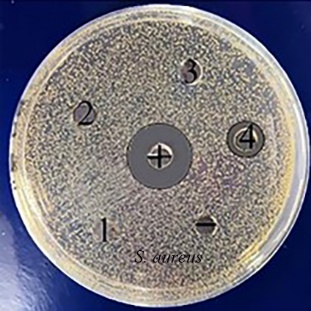 | 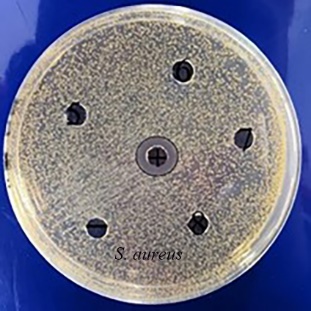 | 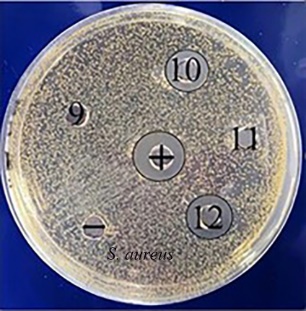 | 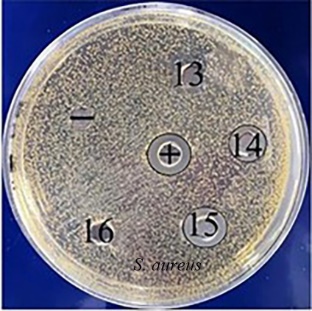 |  |
| **Figure S3D.** The antibacterial effect of peptides FD1-FD16 on *S. aureus* CMCC(B) 26003. The diameter of the Petri dish is 85 mm. The concentration of positive control ampicillin and candidate peptide was 1 mg/mL. *S. aureus* CMCC(B) 26003 at 37°C, cultured for 16-18 h. | | | | |
| 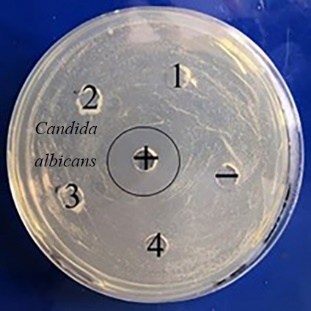 | 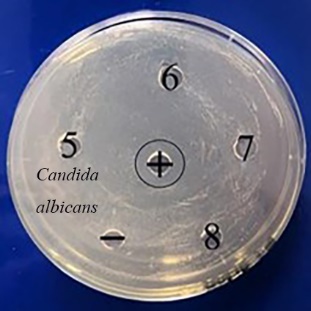 | 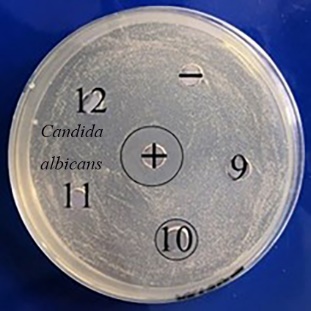 | 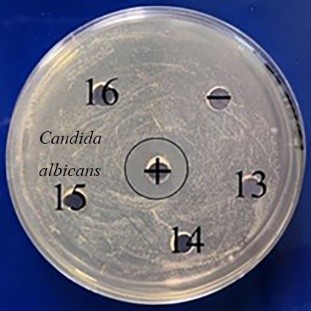 |  |
| **Figure S3E.** The antifungal effect of peptides FD1-FD16 on *C. albicans* CMCC(F) 98001. The diameter of the Petri dish is 85 mm. The concentration of positive control ampicillin and candidate peptide was 1 mg/mL. *C. albicans* CMCC(F) 98001 at 28°C, cultured for 16-18 h. | | | | |

| 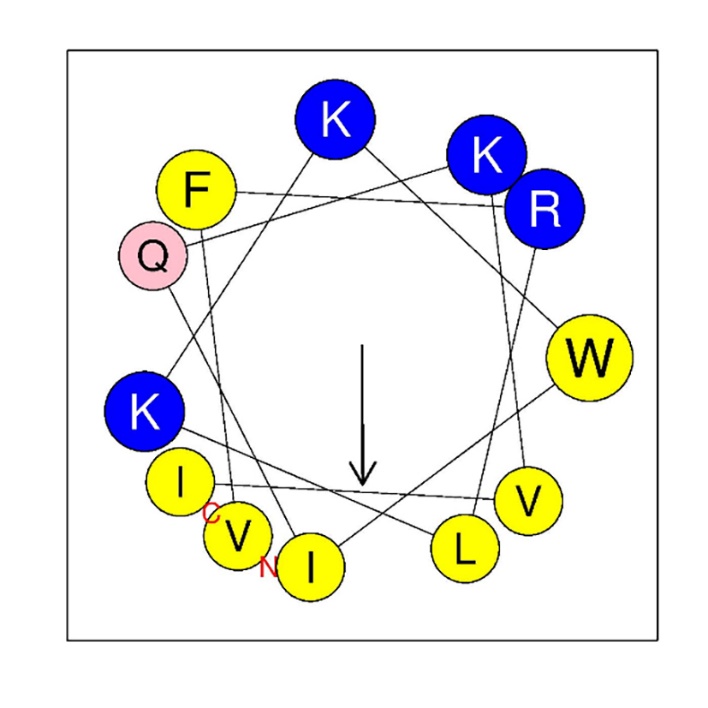  **A** | 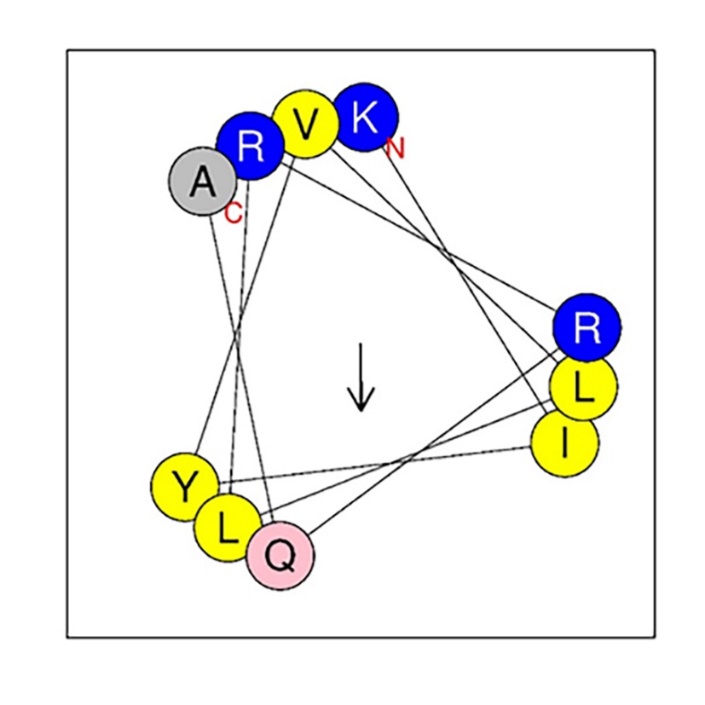  **B** |
| --- | --- |
| 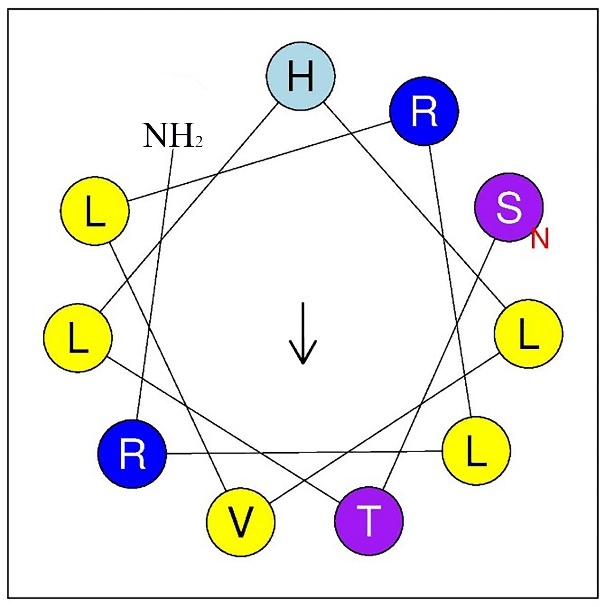  **C** | 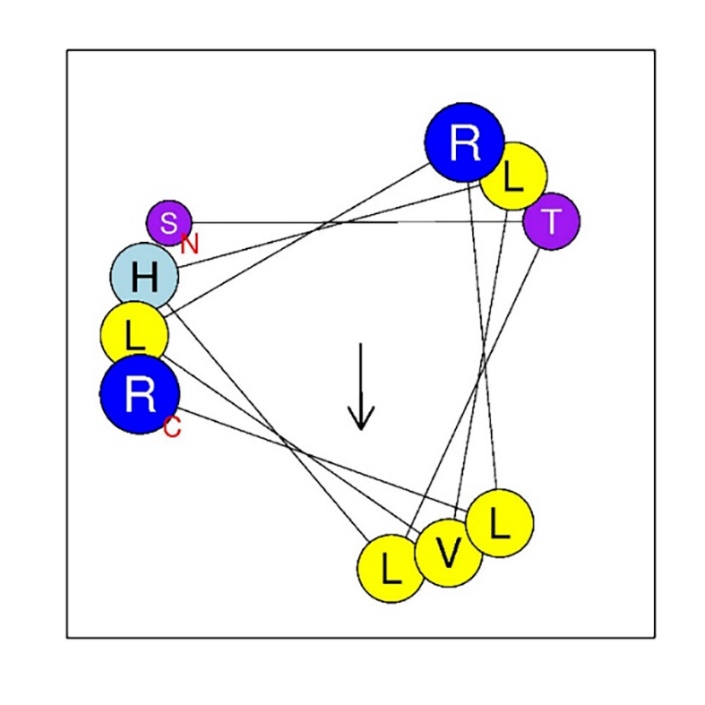  **D** |

**Figure S4. (A)** Helical wheel diagram of the antimicrobial peptide FD10. Hydrophobicity (H): 0.632, Polar residues + GLY (%): 41.67, Hydrophobic moment (µH): 0.610, Nonpolar residues (%): 58.33. **(B)** Helical wheel diagram of the antimicrobial peptide FD12. Hydrophobicity (H): 0.446, Polar residues + GLY (%): 40, Hydrophobic moment (µH): 0.294, Nonpolar residues (%): 60.

**(C)** Helical wheel diagram of the antimicrobial peptide FD15. Hydrophobicity (H): 0.523, Polar residues + GLY (%): 54.55, Hydrophobic moment (µH): 0.263, Nonpolar residues (%): 45.45. **(D)** Helical wheel diagram of the antimicrobial peptide FD16. Hydrophobicity (H): 0.635, Polar residues + GLY (%): 50, Hydrophobic moment (µH): 0.376, Nonpolar residues (%): 50. Dark blue: positively charged residues, yellow: hydrophobic amino acids, blue: basic residues, pink: polar amino acids, arrow: hydrophobic moment, N: N-terminus, C: C-terminus.
